# Comparative Evaluation of the Push-Out Bond Strength and Antimicrobial Efficacy of Biodentine and Bioactive Bone Cement as Root-End Filling Materials: An In Vitro Study

**DOI:** 10.7759/cureus.113972

**Published:** 2026-08-04

**Authors:** Manisha Sharma, Tony Mathew

**Affiliations:** 1 Department of Conservative Dentistry and Endodontics, Vidhya Dental Care, Noida, IND; 2 Department of Conservative Dentistry and Endodontics, A. B. Shetty Memorial Institute of Dental Sciences, Mangaluru, IND

**Keywords:** biodentine, endodontic surgery, enterococcus faecalis, push-out bond strength, root-end filling materials

## Abstract

Introduction: The long-term success of endodontic microsurgery depends on the use of root-end filling materials that provide effective apical sealing while preventing microbial leakage. An ideal retrograde filling material should possess excellent bond strength, biocompatibility, and antimicrobial properties. Biodentine has demonstrated favorable biological and mechanical properties. However, modified bioactive bone cement incorporating mineral trioxide aggregate (MTA) and gentamicin may offer enhanced antibacterial performance. This study compared the push-out bond strength and antimicrobial efficacy of Biodentine and modified bioactive bone cement as root-end filling materials.

Materials and methods: This in vitro experimental study included 60 extracted human mandibular premolars, which were randomly allocated into two groups (n=30): Biodentine and modified bioactive bone cement. Standardized root-end cavity preparation was performed following canal instrumentation and obturation. Push-out bond strength was evaluated using a universal testing machine, while antimicrobial efficacy against *Enterococcus faecalis* was assessed using the agar diffusion method by measuring the zone of inhibition. Data normality was assessed using the Shapiro-Wilk test, and intergroup comparisons were performed using independent-samples t-tests with Welch's correction where appropriate. Statistical significance was established at p<0.05.

Results: Biodentine demonstrated significantly greater push-out bond strength than modified bioactive bone cement (p<0.001). Conversely, modified bioactive bone cement exhibited significantly greater antimicrobial efficacy against *Enterococcus faecalis *than Biodentine (p<0.001). Both comparisons demonstrated large effect sizes, indicating clinically relevant differences between the two materials. These findings suggest that each material possesses distinct advantages in terms of mechanical retention and antibacterial performance.

Conclusion: Under the conditions of this in vitro study, Biodentine demonstrated significantly greater push-out bond strength, whereas modified bioactive bone cement exhibited significantly greater antimicrobial activity against *Enterococcus faecalis*. These findings demonstrate distinct mechanical and antimicrobial properties of the two materials but should not be interpreted as evidence of clinical superiority. Further studies evaluating biocompatibility, sealing ability, long-term material behavior, and in vivo performance are required before the clinical applicability of modified bioactive bone cement can be established.

## Introduction

Successful endodontic treatment depends on the complete elimination of microorganisms from the root canal system, adequate chemomechanical preparation, and the establishment of a durable three-dimensional seal that prevents reinfection [1]. Despite advances in endodontic techniques and materials, treatment failure continues to occur because of persistent microbial contamination, anatomical complexities, procedural errors, or coronal leakage [2]. These factors may result in persistent apical periodontitis, necessitating surgical endodontic intervention when nonsurgical retreatment is either impractical or unlikely to achieve a favorable outcome [3]. Root-end surgery, which includes apicoectomy, root-end cavity preparation, and retrograde filling, aims to eliminate residual infection while creating an effective apical seal that promotes healing of the periapical tissues [4].

The long-term success of endodontic surgery is strongly influenced by the physical, biological, and antimicrobial characteristics of root-end filling materials [5]. An ideal retrograde filling material should provide an excellent seal against microleakage, possess adequate bond strength to resist dislodgement, exhibit dimensional stability, be biocompatible, and demonstrate antibacterial activity against microorganisms commonly associated with persistent endodontic infections [6]. *Enterococcus faecalis *remains one of the most frequently isolated microorganisms from failed root canal treatments because of its ability to survive in harsh environmental conditions, penetrate dentinal tubules, and form resistant biofilms [7]. Consequently, antimicrobial efficacy against *E. faecalis* has become an important criterion for evaluating new root-end filling materials.

Biodentine, a tricalcium silicate-based bioactive cement, has gained widespread acceptance as a root-end filling material owing to its excellent sealing ability, favorable handling characteristics, bioactivity, and capacity to stimulate hard tissue formation [8]. Numerous studies have reported superior push-out bond strength and biocompatibility of Biodentine compared with several conventional retrograde filling materials [9,10]. However, its antibacterial activity has shown variable outcomes in previous investigations, indicating the need for further evaluation.

Polymethyl methacrylate (PMMA)-based bone cement has been extensively used in orthopedic surgery because of its mechanical strength, rapid setting, and ease of manipulation [11]. Nevertheless, conventional PMMA lacks intrinsic bioactivity, limiting its application in endodontics. To overcome this limitation, the present study employed a modified bioactive bone cement prepared by incorporating mineral trioxide aggregate (MTA) and a silane coupling agent into gentamicin-loaded PMMA bone cement [12]. This modification was intended to improve bioactivity while preserving favorable mechanical properties and enhancing antimicrobial performance [13]. The incorporation of gentamicin provides sustained antibacterial activity, whereas MTA contributes bioactive properties that may support periapical healing [12]. Although bioactive bone cement has shown encouraging preliminary results, evidence regarding its bonding performance and antimicrobial effectiveness as a root-end filling material remains limited.

The aim of the present study was to compare the push-out bond strength and antimicrobial efficacy of Biodentine and modified bioactive bone cement as root-end filling materials in an in vitro experimental model. The primary objective was to compare the push-out bond strength of the two materials as an indicator of their ability to resist dislodgement and maintain mechanical stability at the root-end interface. The secondary objective was to evaluate and compare their antimicrobial efficacy against *E. faecalis*, representing their potential to control persistent endodontic infection. By evaluating these complementary mechanical and antimicrobial properties, the study sought to determine whether either material could provide a more favorable combination of mechanical retention and infection control for potential application in endodontic microsurgery.

## Materials and methods

Study design, setting, duration, location, and ethical approval

**Figure 1:**
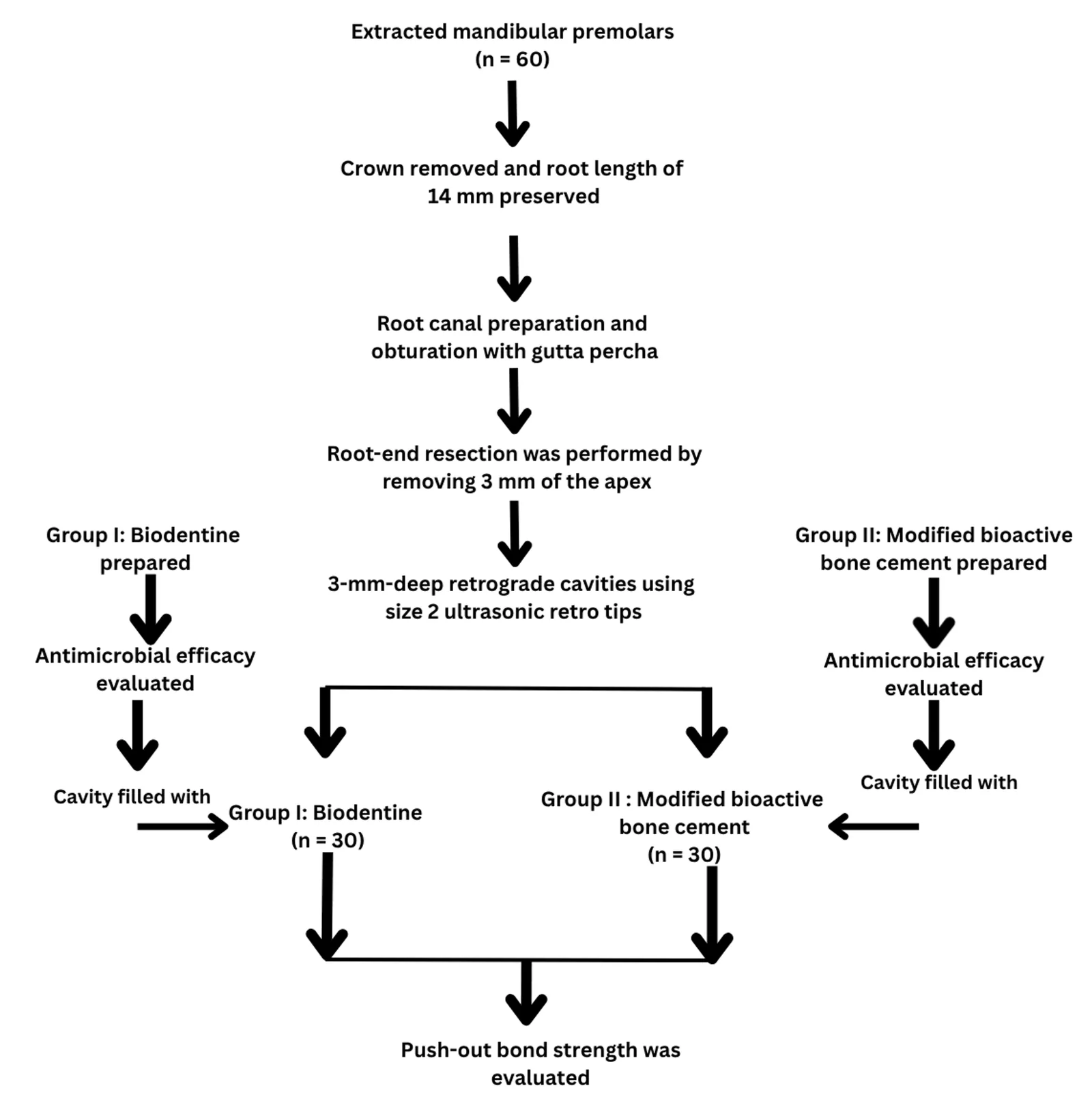
Study flowchart This figure was prepared by the authors using Canva Pro (Canva Pty Ltd., Sydney, Australia).

The present study was designed as an in vitro laboratory-based experimental study and was conducted in the Department of Conservative Dentistry and Endodontics at A. B. Shetty Memorial Institute of Dental Sciences in Mangaluru, Karnataka, India, between May 2023 and December 2023. Ethical approval was obtained from the Institutional Ethical Committee of the college (approval number: ETHICS/ABSMIDS/352/2023; date: April 17, 2023). Extracted human teeth used in the study were obtained with informed consent from patients undergoing orthodontic extractions, and all procedures were performed in accordance with the ethical principles of the Declaration of Helsinki, as revised in 2013 (Figure 1).

Eligibility criteria

Human mandibular premolars extracted for orthodontic reasons were screened for eligibility. Teeth with a single root, a single canal, complete root formation, and canal curvature of <10° (verified on radiograph) were included in the study. Teeth exhibiting multiple roots or canals, caries, cracks, root fractures, root resorption, previous endodontic treatment, calcified canals, incomplete root formation, or canal curvature >10° were excluded to ensure specimen homogeneity and minimize experimental variability.

Standardized experimental protocol

Sixty eligible mandibular premolars were disinfected and stored in normal saline at room temperature. The crowns were removed to obtain a standardized root length of 14 mm. The root canals were prepared using the ProTaper Universal rotary system (Dentsply Maillefer, Ballaigues, Switzerland) up to size F4 with irrigation using 2.5% sodium hypochlorite, normal saline, and 17% ethylenediaminetetraacetic acid (EDTA). The canals were obturated with gutta-percha and AH Plus sealer (Dentsply Maillefer, Ballaigues, Switzerland). Root-end resection was performed by removing 3 mm of the apex using a Mani diamond bur (MANI Inc., Tochigi, Japan), followed by the preparation of 3-mm-deep retrograde cavities using size 2 ultrasonic retro tips (Dentsply Maillefer, Ballaigues, Switzerland).

Following placement and setting of the respective root-end filling materials, each root specimen was sectioned perpendicular to its long axis using a Mani diamond disc (MANI Inc., Tochigi, Japan) mounted on a slow-speed handpiece (NSK Nakanishi Inc., Tochigi, Japan). The sectioning procedure was performed carefully to obtain a transverse root slice containing the root-end filling material at the center of the prepared retrograde cavity. Continuous water cooling was used during sectioning to minimize heat generation and prevent the alteration of the dentin-material interface. Care was taken to maintain the cutting plane perpendicular to the long axis of the root and to avoid excessive pressure, vibration, or damage to the filling material. The obtained sections were inspected for cracks, fractures, voids, or displacement of the root-end filling material before mechanical testing. 

Following root canal obturation, the specimens were stored in individually labeled containers containing normal saline at 37°C for 24 hours to allow complete setting of the root canal sealer. The specimens were subsequently subjected to root-end resection and retrograde cavity preparation. After placement of the respective root-end filling materials, the specimens were maintained at 37°C under humid conditions for 24 hours to permit adequate setting of the materials before sectioning and push-out bond strength testing. Specimens were kept moist throughout the experimental procedure to minimize dehydration-related alterations in dentin.

**Figure 2:**
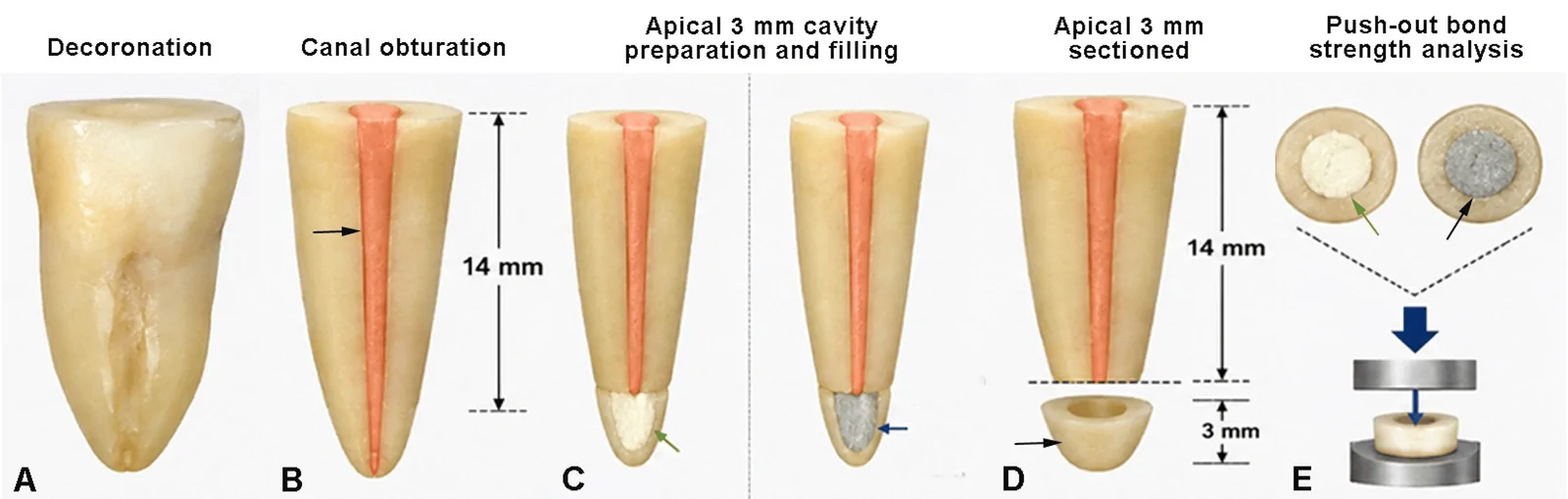
Schematic representation of specimen preparation and push-out bond strength testing (A) Decoronated single-rooted mandibular premolar viewed from the mesial aspect. (B) Root standardized to 14 mm in length and the root canal obturated with gutta-percha (black arrow). (C) Following apical resection and preparation of a 3 mm retrograde cavity, the cavity was filled with either Biodentine (green arrow) or modified bioactive bone cement (blue arrow). (D) The apical 3 mm of the root was sectioned (black arrow) to obtain the test specimen. (E) Transverse sections containing the Biodentine (green arrow) and modified bioactive bone cement (black arrow) were subjected to push-out bond strength analysis. This figure was prepared by the authors using Canva Pro (Canva Pty Ltd., Sydney, Australia).

The specimens were randomly allocated into two groups (n=30 each) using computer-generated simple randomization. A random allocation sequence was generated using the random-number function in Microsoft Excel (Microsoft Corporation, Redmond, Washington, United States), and each specimen was assigned a unique identification code before group allocation. Group I was filled with Biodentine (Septodont, Saint-Maur-des-Fossés, France), whereas Group II received modified bioactive bone cement prepared using Simplex HV bone cement (Stryker Australia Pty Ltd., Sydney, Australia) modified with ProRoot MTA (Dentsply Sirona, Ballaigues, Switzerland) and Monobond-S silane coupling agent (Ivoclar Vivadent AG, Schaan, Liechtenstein). The specimens were embedded in acrylic resin rings, and the push-out bond strength was evaluated using a universal testing machine (Instron 3369, Instron Corp., Norwood, Massachusetts, United States) at a crosshead speed of 0.5 mm/min. For assessment of push-out bond strength, the prepared specimens were identified using coded numbers so that the examiner performing the mechanical testing was blinded to the root-end filling material used. The allocation code was disclosed only after completion and recording of all mechanical measurements. Standardized positioning and loading procedures were maintained for all specimens to minimize measurement-related variability (Figure 2).

For antimicrobial assessment, *E. faecalis* was cultured on Brain Heart Infusion (BHI) agar, and the bacterial suspension was standardized before inoculation [14]. The test materials were placed under identical experimental conditions, and appropriate positive and negative controls were included for assay validation. Gentamicin served as the positive antimicrobial control, whereas a sterile blank without antimicrobial material served as the negative control. The plates were incubated aerobically at 37°C for 24 hours. Antimicrobial activity was determined by measuring the diameter of the zone of inhibition, in millimeters, around each test material. Each experiment was performed in triplicate, and the mean of the three measurements was used for statistical analysis.

Preparation of modified bioactive bone cement

The modified bioactive bone cement was prepared using Simplex HV PMMA bone cement as the base cement. The PMMA-based cement incorporated gentamicin as the antibacterial component and was further modified with ProRoot MTA to impart bioactive properties. Monobond-S silane coupling agent was incorporated to facilitate interaction between the organic PMMA matrix and the added inorganic component. The constituents were mixed thoroughly to obtain a homogeneous consistency before placement. The freshly prepared modified bioactive bone cement was immediately packed into the standardized 3-mm-deep root-end cavities, taking care to achieve complete adaptation to the cavity walls and avoid incorporation of visible voids. The material was subsequently allowed to set before preparation of the specimens for push-out bond strength and antimicrobial testing.

Calibration and reliability

All specimen preparation procedures, retrograde cavity preparation, material placement, and outcome assessments were performed by a single calibrated operator following a standardized experimental protocol to minimize the operator-related variability. Uniform specimen dimensions, instrumentation sequence, root-end cavity depth, testing conditions, and incubation procedures were maintained throughout the study to ensure the reproducibility of the experimental findings.

Sample size calculation

Sample size estimation was performed prospectively using G*Power Version 3.1.9.7 (Heinrich Heine University Düsseldorf, Düsseldorf, Germany) based on a priori power analysis for an independent-samples t-test. The anticipated effect size (Cohen's d=0.70) was derived from Dabbagh et al. [15]. Assuming a two-sided significance level of 5% (α=0.05) and a statistical power of 80% (1−β=0.80), the minimum required sample size was calculated to be 60 specimens. Accordingly, 30 specimens were allocated to each study group to provide adequate statistical power to detect significant differences between the experimental materials.

Statistical analysis

Statistical analysis was performed using IBM SPSS Statistics for Windows, Version 26.0 (IBM Corp., Armonk, New York, United States). Data normality was evaluated using the Shapiro-Wilk test. Continuous variables, including push-out bond strength and antimicrobial zone of inhibition, were expressed as the mean±standard deviation (SD). Intergroup comparisons were performed using the independent-samples t-test, with Welch's correction applied when assumptions of equal variances were violated. Cohen's d was calculated to determine the magnitude of the observed differences between groups. All statistical tests were two-tailed, and p<0.05 was considered statistically significant.

## Results

The comparison of push-out bond strength between the two root-end filling materials is presented in Table 1. Biodentine demonstrated significantly higher mean push-out bond strength than modified bioactive bone cement (101.7±26.8 MPa vs. 60.6±22.2 MPa). The mean difference between the groups was 41.1 MPa (95% CI: 28.37-53.83 MPa), and the difference was statistically significant (t=6.47; p<0.001). The effect size was large (Cohen's d=1.67), indicating a clinically meaningful superiority of Biodentine in terms of dentin adhesion.

**Table 1:** Comparison of push-out bond strength (in megapascals (MPa)) between Biodentine and bioactive bone cement Data are presented as mean±standard deviation (SD). Intergroup comparison was performed using the independent-samples t-test. Effect size was calculated using Cohen's d. *p<0.05 was considered statistically significant.

Group	N	Mean±SD (MPa)	CI at 95% of mean		t-statistic	P-value	Effect size
			Upper	Lower			
Biodentine	30	101.7±26.8	111.71	91.69	6.47	<0.001*	1.67
Bioactive bone cement	30	60.6±22.2	68.89	52.31			

The comparison of antimicrobial efficacy against *E. faecalis* is presented in Table 2. Modified bioactive bone cement exhibited a significantly larger mean zone of inhibition than Biodentine (26.33±1.53 mm vs. 22.67±2.52 mm). The mean difference between the groups was -3.66 mm (95% CI: -4.75 to -2.57 mm), which was statistically significant (t=-6.80; p<0.001). A large effect size (Cohen's d=-1.76) was observed, demonstrating superior antimicrobial activity of modified bioactive bone cement compared with Biodentine.

**Table 2:** Comparison of antimicrobial efficacy (zone of inhibition in mm) between Biodentine and bioactive bone cement Data are presented as mean±standard deviation (SD). Zone of inhibition is expressed in millimeters (mm). Intergroup comparison was performed using the independent-samples t-test. Effect size was calculated using Cohen's d. *p<0.05 was considered statistically significant.

Group	N	Mean±SD (mm)	CI at 95% of mean		t-statistic	P-value	Effect size
			Upper	Lower			
Biodentine	30	22.67±2.52	23.61	21.73	-6.80	<0.001*	1.76
Bioactive bone cement	30	26.33±1.53	26.90	25.76			

## Discussion

The success of endodontic microsurgery depends largely on the ability of the root-end filling material to establish an effective apical seal while preventing bacterial recolonization [1]. Consequently, both mechanical stability and antimicrobial activity are considered essential characteristics of an ideal retrograde filling material [6]. The present in vitro study compared Biodentine and a modified bioactive bone cement in terms of push-out bond strength and antimicrobial efficacy against* E. faecalis*. The findings demonstrated that Biodentine exhibited significantly greater push-out bond strength, whereas the modified bioactive bone cement showed significantly superior antimicrobial activity.

The push-out bond strength is an important indicator of the resistance of a root-end filling material to dislodgement under functional and clinical stresses. In the present study, Biodentine demonstrated significantly higher bond strength than modified bioactive bone cement. This superior performance can be attributed to the tricalcium silicate composition of Biodentine, which promotes biomineralization and the formation of hydroxyapatite crystals at the dentin-material interface [8]. The release of calcium ions facilitates the development of an interfacial mineral infiltration zone and tag-like structures within the dentinal tubules, thereby improving micromechanical interlocking and adhesion. Furthermore, the fine particle size and hydrophilic nature of Biodentine allow enhanced penetration into the dentinal tubules and adaptation to the cavity walls, contributing to its greater resistance to dislodgement [8].

These findings are consistent with those of Parmar et al. [9], who reported a significantly greater push-out bond strength for Biodentine than for other calcium silicate-based repair materials. Similarly, Guneser et al. [10] demonstrated that Biodentine maintained superior bond strength even after exposure to various endodontic irrigants, highlighting its stable adhesion properties. Atmeh et al. [16] further explained that the chemical interaction between calcium silicate cements and dentin results in biomineralization and the formation of an acid-resistant interfacial layer that enhances bond durability. Collectively, these studies support the superior mechanical performance of Biodentine observed in the present study.

In contrast, the modified bioactive bone cement exhibited significantly greater antimicrobial activity against *E. faecalis* than Biodentine. Persistent infection by *E. faecalis* is a major cause of endodontic treatment failure because of its ability to survive under nutrient-deprived conditions, invade dentinal tubules, and form resistant biofilms [7]. Therefore, the antibacterial activity of root-end filling materials is of considerable clinical importance. The enhanced antimicrobial efficacy observed in the present study is primarily attributable to the incorporation of gentamicin within the PMMA-based bone cement. Gentamicin is a broad-spectrum aminoglycoside antibiotic that provides sustained local antimicrobial release while maintaining high concentrations at the application site without significant systemic exposure [17]. In addition, the incorporation of mineral trioxide aggregate into the bone cement may have contributed to an alkaline environment that further inhibited bacterial growth [18].

These findings are consistent with those of Chang et al. [19], who demonstrated the excellent antimicrobial performance of gentamicin-loaded PMMA bone cement for infection control in orthopedic applications. Chen et al. [20] reported sustained antibiotic release from modified PMMA bone cement with prolonged antibacterial efficacy. Although previous investigations by Esteki et al. [14] demonstrated the antimicrobial activity of Biodentine, the present study found that the modified bioactive bone cement produced significantly larger zones of inhibition. This difference is likely due to the sustained release of gentamicin, which provides a stronger antibacterial effect than the indirect antimicrobial action of calcium silicate cements, which mainly depends on hydroxyl ion release and alkalinity.

An important aspect of the present investigation is the development and evaluation of a modified bioactive bone cement by incorporating ProRoot MTA and a silane coupling agent into conventional PMMA bone cement. While previous studies have investigated PMMA bone cement in orthopedic applications, evidence regarding its use as a root-end filling material remains limited [11,19]. Therefore, the present study provides preliminary evidence that modification of PMMA bone cement can substantially improve its biological performance while maintaining clinically acceptable mechanical properties. Although its push-out bond strength remained lower than that of Biodentine, the values obtained were sufficient to suggest potential clinical applicability, particularly in situations where enhanced antimicrobial activity is desirable.

The findings indicate that material selection should be guided by clinical objectives. Biodentine remains a suitable choice when maximum adhesion and resistance to dislodgement are required during surgical endodontic treatment. Conversely, modified bioactive bone cement may be advantageous in cases of persistent periapical infection because of its superior antibacterial properties. Further optimization of the formulation may improve its bonding characteristics while preserving its antimicrobial efficacy.

Clinical implications and limitations

The present findings suggest that Biodentine is preferable when superior mechanical retention and sealing ability are the primary clinical requirements, whereas modified bioactive bone cement may be considered a promising alternative in infected surgical sites because of its enhanced antibacterial activity. However, the study was conducted under controlled laboratory conditions that do not fully reproduce the complex oral environment, including blood contamination, tissue fluid, functional loading, and host immune responses. Only the immediate material properties were evaluated, and long-term durability, sealing ability, bioactivity, cytocompatibility, ion release, and clinical performance were not assessed. Therefore, well-designed animal studies followed by long-term randomized clinical trials are required before modified bioactive bone cement can be recommended for routine clinical use.

## Conclusions

Within the limitations of this in vitro study, Biodentine demonstrated significantly greater push-out bond strength, whereas modified bioactive bone cement exhibited significantly greater antimicrobial activity against *E. faecalis*. These findings indicate that the two materials possess distinct mechanical and antimicrobial properties under the experimental conditions evaluated. However, the present results are limited to push-out bond strength and agar diffusion-based antimicrobial assessment and should not be extrapolated directly to clinical performance. Further investigations evaluating sealing ability, biocompatibility, bioactivity, long-term durability, and in vivo performance are necessary to determine the potential clinical applicability of modified bioactive bone cement as a root-end filling material.

## References

[1] Mehta D, Coleman A, Lessani M (2025). Success and failure of endodontic treatment: predictability, complications, challenges and maintenance. Br Dent J.

[2] Tabassum S, Khan FR (2016). Failure of endodontic treatment: the usual suspects. Eur J Dent.

[3] Falatah AM, AlQari AA, Alrwiali MA (2025). Clinical outcomes of nonsurgical retreatment in teeth with persistent apical periodontitis: a systematic review. Cureus.

[4] von Arx T (2011). Apical surgery: a review of current techniques and outcome. Saudi Dent J.

[5] Patil P, Gupta A, Kishlay K, Rathaur S, Vaidya N, Sharma M, Gupta S (2025). Comparative evaluation of the antimicrobial efficacy of endodontic sealers against Staphylococcus aureus and Streptococcus mutans: an in vitro study. Cureus.

[6] Džanković A, Hadžiabdić N, Korać S, Tahmiščija I, Konjhodžić A, Hasić-Branković L (2020). Sealing ability of mineral trioxide aggregate, Biodentine and glass ionomer as root-end materials: a question of choice. Acta Med Acad.

[7] AlShwaimi E, Bogari D, Ajaj R, Al-Shahrani S, Almas K, Majeed A (2016). In vitro antimicrobial effectiveness of root canal sealers against Enterococcus faecalis: a systematic review. J Endod.

[8] Malkondu Ö, Karapinar Kazandağ M, Kazazoğlu E (2014). A review on Biodentine, a contemporary dentine replacement and repair material. Biomed Res Int.

[9] Parmar S, Aggarwal N, Gupta H, Marwaha J, Pundir P, Saini R (2024). Comparative evaluation of the push-out bond strength of glass ionomer cement, mineral trioxide aggregate, Biodentine, and endosequence root repair material in repair of furcation perforations: an in vitro study. J Pharm Bioallied Sci.

[10] Guneser MB, Akbulut MB, Eldeniz AU (2013). Effect of various endodontic irrigants on the push-out bond strength of Biodentine and conventional root perforation repair materials. J Endod.

[11] Ramanathan S, Lin YC, Thirumurugan S, Hu CC, Duann YF, Chung RJ (2024). Poly(methyl methacrylate) in orthopedics: strategies, challenges, and prospects in bone tissue engineering. Polymers (Basel).

[12] Miyazaki T, Ohtsuki C, Kyomoto M, Tanihara M, Mori A, Kuramoto K (2003). Bioactive PMMA bone cement prepared by modification with methacryloxypropyltrimethoxysilane and calcium chloride. J Biomed Mater Res A.

[13] Verné E, Bruno M, Miola M, Maina G, Bianco C, Cochis A, Rimondini L (2015). Composite bone cements loaded with a bioactive and ferrimagnetic glass-ceramic: leaching, bioactivity and cytocompatibility. Mater Sci Eng C Mater Biol Appl.

[14] Esteki P, Jahromi MZ, Tahmourespour A (2021). In vitro antimicrobial activity of mineral trioxide aggregate, Biodentine, and calcium-enriched mixture cement against Enterococcus faecalis, Streptococcus mutans, and Candida albicans using the agar diffusion technique. Dent Res J (Isfahan).

[15] Dabbagh NK, Esnaashari E, Bakhtiar H, Nekoofar MH, Ghezelsofla M (2021). In vitro comparison of pushout bond strength of ProRoot MTA, Biodentine and TheraCal. J Clin Exp Dent.

[16] Atmeh AR, Chong EZ, Richard G, Festy F, Watson TF (2012). Dentin-cement interfacial interaction: calcium silicates and polyalkenoates. J Dent Res.

[17] Sohail MR, Esquer Garrigos Z, Elayi CS, Xiang K, Catanzaro JN (2020). Preclinical evaluation of efficacy and pharmacokinetics of gentamicin containing extracellular-matrix envelope. Pacing Clin Electrophysiol.

[18] Estrela C, Cintra LT, Duarte MA, Rossi-Fedele G, Gavini G, Sousa-Neto MD (2023). Mechanism of action of bioactive endodontic materials. Braz Dent J.

[19] Chang Y, Tai CL, Hsieh PH, Ueng SW (2013). Gentamicin in bone cement: a potentially more effective prophylactic measure of infection in joint arthroplasty. Bone Joint Res.

[20] Chen L, Tang Y, Zhao K, Zha X, Liu J, Bai H, Wu Z (2019). Fabrication of the antibiotic-releasing gelatin/PMMA bone cement. Colloids Surf B Biointerfaces.

